# Extraction and Quantification of Bioactive Tyrian Purple Precursors: A Comparative and Validation Study from the Hypobranchial Gland of a Muricid *Dicathais orbita*

**DOI:** 10.3390/molecules21121672

**Published:** 2016-12-05

**Authors:** Roselyn Valles-Regino, Peter Mouatt, David Rudd, Lachlan H. Yee, Kirsten Benkendorff

**Affiliations:** 1 Marine Ecology Research Centre, School of Environment, Science and Engineering, Southern Cross University, Lismore, NSW 2480, Australia; r.regino.10@student.scu.edu.au (R.V.-R.); david.rudd@scu.edu.au (D.R.); lachlan.yee@scu.edu.au (L.H.Y.); 2 Southern Cross Plant Science, Southern Cross University, Lismore, NSW 2480, Australia; peter.mouatt@scu.edu.au

**Keywords:** brominated indole, marine natural products, nutraceutical, marine mollusc, liquid chromatography–mass spectrometry

## Abstract

Muricidae are marine molluscs known for the production of Tyrian purple and bioactive precursor compounds. A validation study for the extraction and analysis of secondary metabolites found in the hypobranchial gland of the muricid *Dicathais orbita* is reported, using high performance liquid chromatography–mass spectrometry (HPLC-MS) with diode array detector (DAD). Quantification of the dominant secondary metabolites from *D. orbita* is described, followed by a comparison of solvent extraction procedures and stability studies. The intra- and inter-day relative standard deviation (RSD) for tyrindoxyl sulphate was 0.46% and 0.17%, respectively. The quantification was linear for standards murexine, 6-bromoisatin, and tyrindoxyl sulphate. The limits of detection were 0.03, 0.004, and 0.07 mg/mL, respectively, and the limits of quantification were 0.09, 0.01, and 0.22 mg/mL, respectively. The results showed that alcoholic solvents were better for extracting choline ester and indoxyl sulphate ultimate precursors, while chloroform was more suitable for the extraction of the intermediate precursors. Multivariate analysis revealed significant differences in extract composition according to the solvent used. Stability testing showed an increase of the oxidative compounds 6-bromoisatin and putative tyrindoxyl S-oxide sulphate in the ethanol extracts while more degradation products were seen in the chloroform extracts after months of cold storage. The validated method was found to be simple, reproducible, precise, and suitable for quantification of the secondary metabolites of muricid molluscs for dye precursor and nutraceutical quality control, as well as applications in marine chemical ecology.

## 1. Introduction

Marine invertebrates are the source of thousands of unique compounds featuring different structural classes and functional groups, including a relatively high incidence of halogenation [1]. Marine organisms produce many novel biologically active compounds with potential therapeutic applications, including anticancer, antimicrobial, antibacterial, and cytotoxic activities [2]. A few nutraceuticals (natural health products) from marine sources are commercially available, such as squalamine from dogfish shark [3], Lyprinol^®^ from the New Zealand green-lipped mussel [4] and fish oils containing omega-3 fatty acids eicosapentaenoic acid (EPA) and docosahexaenoic acid (DHA) [5]. The Muricidae family of predatory marine gastropods have been used traditionally as a source of historically important dye pigments and natural medicines [6,7]. Recent research has revealed that these molluscs produce several biologically active compounds with the potential for further development as nutraceuticals [6,8]. Quality control of new marine natural products, such as Muricidae extracts, for commercial use and clinical trials, requires analytical methods that are reproducible, with validated quantification capacity.

Herbal extracts that are registered as therapeutic goods typically have validated analytical methods to help monitor the safety, quality and efficacy of the products, in accordance with legislative requirements for Good Manufacturing Practice (GMP). Validation of analytical methods is a process of establishing standardised procedures for repeated use, with a high degree of assurance that reliable data can be obtained to accurately assess the quality of natural extracts and the relative quantity of the analytes of interest [9]. The parameters considered during the validation process include method selection, optimisation, selectivity, robustness, precision, and stability, among others [10]. The goal of obtaining a fully validated method for the analysis of new marine natural products may be a challenge, but an inadequate approach will result in wasted time, money, and resources [11].

**Figure 1:**
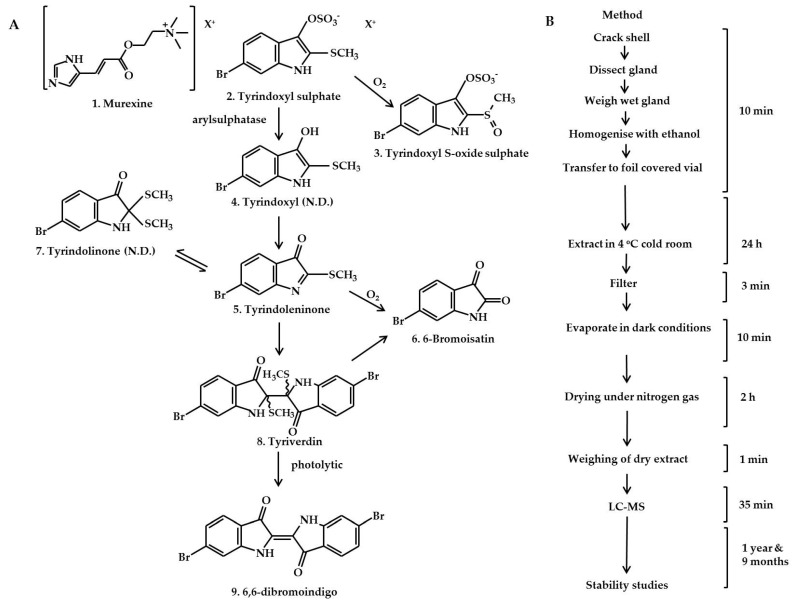
Extraction of precursors and generation pathway of Tyrian purple detected by LC-MS from the hypobranchial gland of *D. orbita*: (**A**) Reaction scheme for generation of Tyrian purple from precursors and (**B**) the extraction procedure showing the time spent for each step in the preparation of extract. N.D.—not detected.

Muricidae molluscs are known to produce two classes of bioactive compounds: polar choline esters (e.g., murexine **1**, Figure 1A) and hydrophilic (**2** and **3**, Figure 1A) and lipophilic brominated indoles (**4**–**9**, Figure 1A). These compounds are stored together as indoxyl sulphate salts, with different non-brominated and brominated precursors produced by different Muricidae species [6,7]. These ultimate precursor salts are hydrolysed by an aryl sulphatase enzyme, after which they undergo a series of spontaneous and oxidative reactions to yield intermediate dye precursors including tyrindoxyl (**4**, Figure 1A), tyrindoleninone (**5**, Figure 1A), 6-bromoisatin (**6**, Figure 1A), tyrindolinone (**7**, Figure 1A), and tyriverdin (**8**, Figure 1A). Photolytic cleavage of tyriverdin produces 6,6′-dibromoindigo (**9**, Figure 1A), the main pigment in the famous and expensive dye, Tyrian purple [7]. The brominated indole precursors have demonstrated in vitro and in vivo anti-cancer properties [8,12,13,14], as well as antibacterial activity [15,16]. Muricid extracts containing these brominated indoles appear to have low toxicity in rodent models [14,17] suggesting the potential to develop a safe and effective nutraceutical extract. On the other hand, the choline esters have muscle relaxing and pain-relieving activities [18], but can exhibit some toxic side-effects in vivo [6]. In particular, tigloycholine from the muricid *Thais clavigera* was found to be over six times more toxic than murexine, causing convulsions and eventually death in mice when administered intravenously, within two minutes at <1 mg/kg [19]. In consideration of this, it is necessary to monitor the ultimate precursor composition in Muricidae extracts intended for nutraceutical development due to potential safety concerns.

By far, the most useful tool for the separation and monitoring of complex mixtures of small molecules is high-performance liquid chromatography (HPLC) [20]. Coupled with mass spectroscopy (MS), LC-MS has been found to be efficient for detailed multi-component analyses of the extracts from Muricidae [21]. Westley and Benkendorff [21] adapted an HPLC-MS approach to qualitatively analyse brominated precursors and pigments composing Tyrian purple from the hypobranchial and reproductive glands of Muricidae. To maximise the extraction of the final dye pigments, hot dimethylformamide (DMF) was used and, although some polar tyrindoxyl sulphate (**2**, Figure 1) was detected, no choline esters were recovered using this extraction method [21]. A number of other studies testing the anti-cancer properties have used chloroform extracts to concentrate the intermediate brominated indole dye precursors [12,13,22,23] and again LC-MS analysis confirmed the absence of any choline esters in these extracts. Chloroform [24] and DMF [25] are toxic solvents and are therefore not recommended for the preparation of nutraceuticals for human use. As all intermediate bioactive compounds and dye pigments can be produced by controlling the reactions from polar precursors, a reproducible extraction method using less toxic alcoholic solvents is recommended to extract the full range of bioactive secondary metabolites in Muricidae. This will facilitate future metabolomics studies on the ultimate precursors produced by these molluscs and establish some parameters that will be useful for quality control of Muricidae-derived medicinal products.

*Dicathais orbita* is an Australian species of Muricidae that has been established as a good model for natural product research [8]. This species only produces a single brominated indole precursor, tyrindoxyl sulphate (**2,** Figure 1A) [7], but both murexine (**1**, Figure 1A) and senecioycholine have also been reported from the hypobranchial glands [18]. In the present work, a comparative study was undertaken using different extraction solvents and detection using HPLC-electrospray ionisation (ESI)-MS and chemical ionisation (CI)-MS techniques, in positive and negative modes, for quantifying the secondary metabolites found in the hypobranchial gland of *D. orbita*. The preparation of the extracts was undertaken using standardised conditions to minimise artefacts (Figure 1B). Validation studies were performed employing parameters to establish precision, linearity, and limits of detection and quantification. Stability testing was also undertaken to determine any oxidative and photolytic degradation of the main precursors under different storage conditions. This study provides a procedure for the routine extraction and analysis of the low molecular weight secondary metabolites produced and stored in the hypobranchial glands of *D. orbita* for future nutraceutical development and chemical ecology research. In light of the rising interest in marine-derived bioactive compounds [26], this study provides a good precedent for developing strategies for marine natural product extraction, with both qualitative and quantitative analyses.

## 2. Results and Discussion

### 2.1. Identification of the Bioactive Compounds in the Extracts

**Figure 2:**
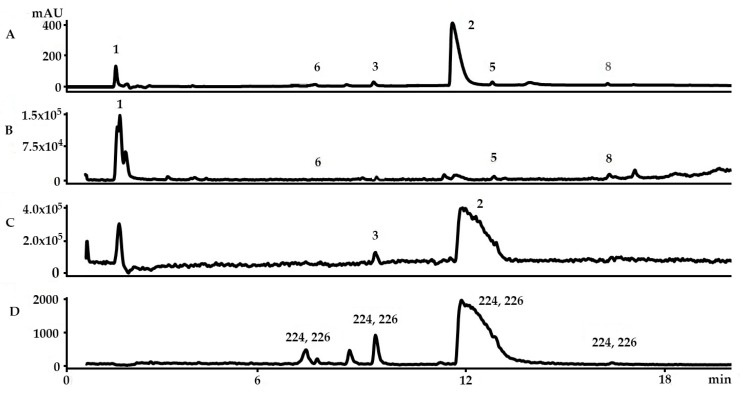
Liquid chromatography–mass spectrometry analysis of an ethanol extract from the *Dicathais orbita* hypobranchial gland: (**A**) high performance liquid chromatography chromatogram at 210 nm; (**B**) total ion current (TIC) in positive ion mode; (**C**) TIC in negative mode; and (**D**) selected ion monitoring (SIM) for 224 and 226 in equal ratios for bromoisatin isotopic fragment ions Br^79^ Br^81^. Numbered peaks based on Figure 1 correspond to (**1**) murexine, (**2**) tyrindoxyl sulphate, (**3**) tyrindoxyl S-oxide sulphate, (**5**) tyrindoleninone, (**6**) 6-bromoisatin, and (**8**) tyriverdin.

As ethanol is the safest solvent for the extraction of natural products for human use and suitable for the polar precursors of Tyrian purple, this solvent was used as the basis for comparative analysis of the secondary metabolites that can be extracted from the hypobranchial glands of *D. orbita*. Chemical analysis by LC-MS confirmed the presence of both brominated indole and choline ester precursors in the ethanol extracts (Figure 2). Previous attempts to analyse *D. orbita* extracts by LC-MS have not simultaneously detected murexine with the brominated indoles [13,21,27]. Six peaks were detected in the ethanol extract by HPLC-DAD at 210 nm (Figure 2A) and four of these were detected by ESI-MS in positive mode: murexine (**1**), 6-bromoisatin (**6**), tyrindoleninone (**5**), and a sodium adduct of tyriverdin (**8**) (Figure 2B); whereas the two remaining compounds tyrindoxyl sulphate (**2**) and its oxidation product, tentatively identified as tyrindoxyl S-oxide sulphate (6-bromo-2-methylsulfinyl-indoxyl-3-sulfate) (**3**), were only detected as negative ions (Figure 2C). We obtained similar results using CI-MS, with murexine and the same five brominated indoles detected (Supplementary Figure S1), although ESI-MS was more sensitive for the determination of tyriverdin (Figure 2). Consequently, ESI-MS is recommended and should be routinely run in both positive and negative mode to identify the full suite of Tyrian purple precursors.

**Figure 3:**
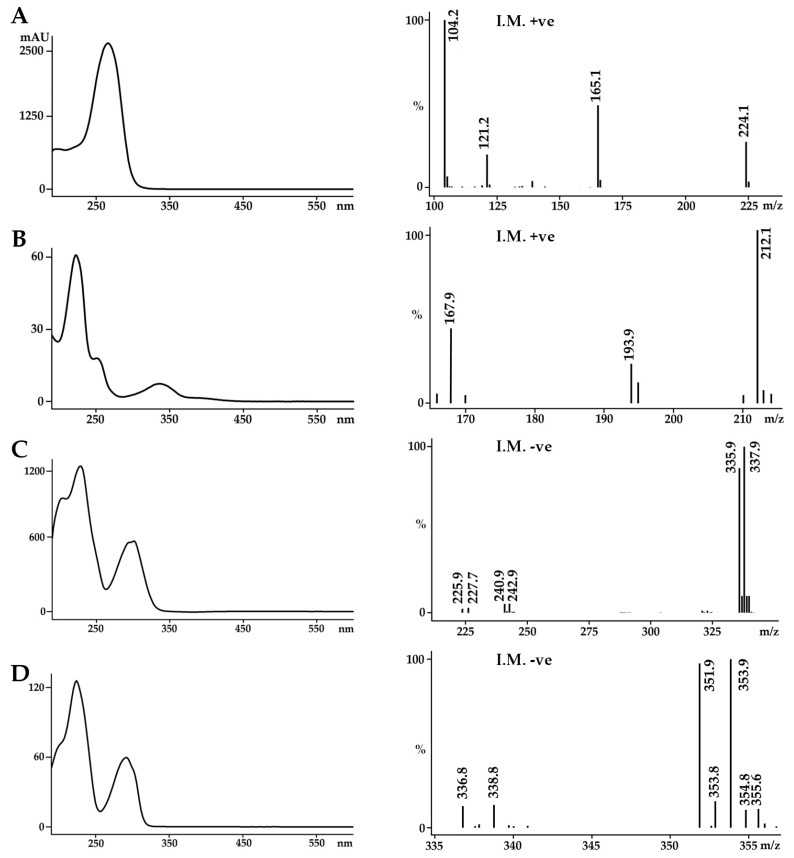
UV spectra (left), and mass spectra (right) for the ultimate precursors of Tyrian purple found in the hypobranchial gland of *Dicathais orbita* from high-performance liquid chromatography-mass spectrometry (HPLC-MS) with electrospray ionisation (ESI). The compounds are: (**A**) murexine (**1**); (**B**) indoxyl sulphate; (**C**) tyrindoxyl sulphate (**2**); and (**D**) tyrindoxyl S-oxide sulphate (**3**). I.M. ionisation mode: I.M. +ve = positive ions, I.M. −ve = negative ions.

**Figure 4:**
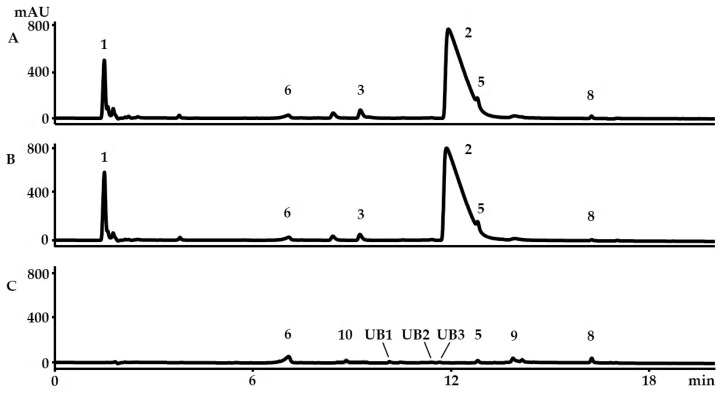
Representative chromatograms of the extracts from the hypobranchial gland of *Dicathais orbita* from the diode array detector at 210 nm. The solvents used for the extraction of the secondary metabolites are: (**A**) methanol; (**B**) ethanol; and (**C**) chloroform. Numbered peaks are as follows: (**1**) murexine, (**2**) tyrindoxyl sulphate, (**3**) tyrindoxyl S-oxide sulphate, (**5**) tyrindoleninone, (**6**) 6-bromoisatin, (**8**) tyriverdin, (**9**) 6,6-dibromoindigo, (**10**) indoxyl sulphate, and unidentified brominated indoles UB1, UB2, and UB3.

Selected ion monitoring (SIM) at *m*/*z* 224, 226 was highly effective for detecting all the brominated indole precursors with increased sensitivity (Figure 2D), based on equal intensity for Br^79^, Br^81^ of a bromoisatin fragment ion. The brominated indoles detected using this SIM approach in the LC-MS have all been previously described as secondary metabolites in the hypobranchial gland of *D. orbita* [8,13,28,29]. Murexine (**1**), also a known secondary metabolite of *D. orbita*, has a molecular ion at 224 (Figure 3A), but, as expected, this compound was not detected by single ion monitoring (Figure 2D) due to the lack of bromine for paired isotopic mass at 226. Murexine occurs as a cluster of peaks between *t*_R_ 1.4–1.6 min (Figure 2A,B), all with a mass ion at *m*/*z* 224, fragment ions at *m*/*z* 104, 121, 165, and UV λ_max_/nm of 266 (Figure 3A). This is consistent with the mass spectra previously reported for murexine [27], with the different HPLC peaks likely to represent different structural isomers of the same compound. The fraction containing these peaks was collected and confirmed as murexine by TLC and ^1^H-NMR (Table 1). Purified murexine was resuspended in deuterated acetonitrile (CD_3_CN) for ^1^H-NMR, where all proton shifts were detected except the NH on the imidazole ring, which is often lost from intermolecular exchange or shielding [30]. Although murexine occurred as a large peak (Figure 2A and Figure 4A,B), tigloycholine (*m*/*z* 186), which has been identified in other muricids and is reported to be more potent than murexine [19], was not detected in any of the samples. Senecioylcholine, an isomer of tigloycholine that has been previously reported in significant quantities together with murexine in *D. orbita* [18], was also not detected.

Tyrindoxyl sulphate, the ultimate precursor of Tyrian purple in *D. orbita*, was identified as the most intense peak in the HPLC at 210 nm, TIC in negative mode, and SIM (Figure 2A,C,D). The molecular mass of this peak corresponded to tyrindoxyl sulphate, *m*/*z* 337, 339 (Br^79^ Br^81^) [M + H]^+^ (Figure 3C). This was consistent with previous studies by Westley and Benkendorff [21] and Esmaeelian et al. [13], although tyrindoxyl sulphate appeared earlier at *t*_R_ 5.1–5.5 min and 8.58 min, respectively, in those studies due to differences in the elution schemes. The identification of this ultimate precursor was further confirmed by ^1^H-NMR (Table 1) after preparative HPLC. Tyrindoxyl sulphate was resuspended in deuterated water (D_2_O), and identified based on the proton shifts located on the benzene ring of the indole structure and the S-methyl group, with the NH not reported due to the field shift often encountered from shielding effects [7]. In LC-MS, at a higher concentration, tyrindoxyl sulphate tended to drag on the column and overlapped with tyrindoleninone (Figure 4A,B); however, these co-eluting compounds can be detected in different ion modes (Figure 2B,C). On the contrary, at a lower concentration, this dominant compound was seen to elute earlier than the tyrindoleninone (Figure 2A). Evidence for a second ultimate precursor in the ethanol extracts of *D. orbita* was found in a minor peak at 8.6 min (Figure 2C), which produced a strong signal in ESI-MS negative mode at *m*/*z* 352, 354 (Figure 3D) and is tentatively identified as tyrindoxyl S-oxide sulphate. The salt of 6-bromo-2-methylsulfonylindoxylsulfate has been previously predicted by IR spectrophotometry from the hypobranchial gland of the Muricidae *Plicopurpura pansa* [31]. Both of these brominated indoxyl sulphate precursors have similar UV profiles with λ_max_ around 225 and 300 nm (Figure 3C,D). Previously in the literature, tyrindoxyl S-dioxide has been reported in muricids [7,32], but there has been no previous report of a derivative for a single oxygen such as the one detected in our samples and it is quite likely to be an artefact from alcoholic extraction and/or oxidation.

**Figure 5:**
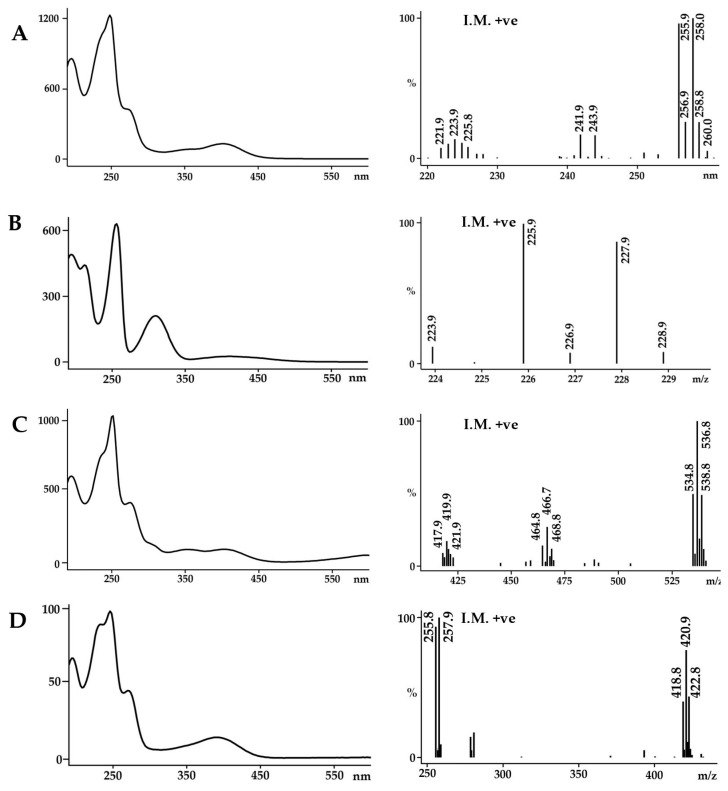
UV spectra (**left**) and mass spectra (**right**) for the intermediate precursors and end product found in the hypobranchial gland of *Dicathais orbita* from high performance liquid chromatography-mass spectrometry (HPLC-MS) with electrospray ionisation (ESI). The compounds are: (**A**) tyrindoleninone (**5**); (**B**) 6-bromoisatin (**6**); (**C**) tyriverdin (**8**); and (**D**) 6,6′-dibromoindigo (**9**). I.M. ionisation mode: I.M. +ve = positive ions, I.M. −ve = negative ions.

**Figure 6:**
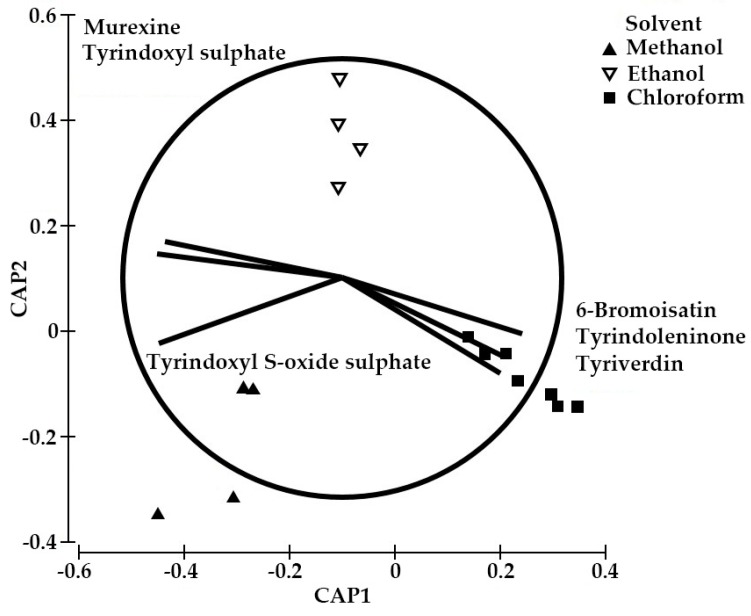
Canonical analysis on the principal coordinates (CAP) based on the secondary metabolites from the hypobranchial gland of *Dicathais orbita* detected in different organic solvents, with vector overlay based on Spearman correlation > 0.6.

The intermediate monobrominated and dibrominated precursors of Tyrian purple can be identified by their characteristic duplet and triplet isotopic ion clusters in the MS [21]. Consistent with previous research, we detected tyrindoleninone (Figure 5A) and 6-bromoisatin (Figure 5B) by ESI-MS in the ethanol extracts of *D. orbita*, with pseudomolecular ions [M + H]^+^ 256, 258 and [M + H]^+^ 226, 228, respectively, for Br^79^, Br^81^. Adducts of cations (e.g., [M + NH_4_]^+^, [M + Na]^+^, [M + K]^+^) are possible in MS [33], especially when the specimens are collected from seawater, where high cation concentrations are present. The sodium ion of tyrindoxyl sulphate, for example, has been previously isolated from *D. orbita* by Baker and Duke [34]. Another minor peak at 16.3 min (Figure 2A) registered a dibrominated isotopic cluster at *m*/*z* 537, 539, 541 (Figure 5C), which corresponds to the sodium ion adduct of tyriverdin ([M + Na]^+^ Br^79^ Br^79^, Br^79^ Br^81^, Br^81^ Br^81^). Baker and Sutherland [35] were also able to isolate tyriverdin and described it as a pale green, light-sensitive solid that is formed from the reaction of tyrindoxyl and tyrindoleninone. The chemical structure of tyriverdin was corrected by Christophersen et al. [36] based on the molecular fragment ions from field desorption/field ionisation mass spectrometry and ^1^H-NMR analysis. Benkendorff et al. (2000) [15] investigated this compound from the egg masses of *D. orbita* using gas chromatography-MS in both ESI-MS and CI-MS positive mode, but obtained only the [M − 2SCH_3_], as the two methyl thio (SCH_3_) groups were lost under electron impact in the MS. The molecular ion for tyriverdin was detected for the first time in *D. orbita* using HPLC-ESI-MS by Westley and Benkendorff and was found in the hypobranchial and capsule glands of females [21], but this compound was not detected by MS imaging after tissue imprinting on nanostructured surfaces [29].

One of the other transient compounds, tyrindolinone (**7**, Figure 1A), has been previously detected in lipophilic extracts from *D. orbita* hypobranchial glands by LC-MS [21] and nano-assisted laser desorption/ionisation (NALDI)-MS [29], but was absent in our extracts. This may be because the formation of tyrindolinone requires reducing conditions and is easily oxidised in air [7]. Secondly, the mercaptan or methane thiol fragment in tyrindolinone is readily lost during chromatography and heating to yield the more stable tyrindoleninone [7]. Thirdly, polar solvents such as ethanol may have precluded the extraction of hydrophobic tyrindolinone. Fourth, by scaling down the sample sizes and solvent volumes and using a more rapid extraction and drying procedure (Figure 1B), we may have impeded the formation of this compound. Finally, there is a possibility that one of the unknowns was tyrindolinone but did not produce a clear molecular ion or did not separate well from one of the other precursors in our system. Overall, the immediate precursors of Tyrian purple remain relatively difficult to detect due to insolubility and their susceptibility to degradation in light, oxygen, and under electron impact. However, tyriverdin and the related brominated indole derivatives produce a characteristic UV trace in HPLC-DAD, with λ_max_ around 250 nm and a broad low peak in the UVA-visible light spectrum between 350 and 450 nm (Figure 5A–D).

The only known compounds not detected in ethanol and methanol extracts, but present in the chloroform extract of *D. orbita*, were indoxyl sulphate (Figure 3B) and 6,6′-dibromoindigo (Figure 5D). The non-brominated indoxyl sulphate precursor of indigo and indirubin has not been previously identified in *D. orbita* hypobranchial glands [7] and egg masses [16]. However, indoxyl sulphate has been reported in the secretions from the hypobranchial glands of *Trunculariopsis* (*Murex*) *trunculus* [32] and the non-brominated pigments have been detected in several Muricidae species [7,37]. Despite the detection of indoxyl sulphate in *D. orbita* for the first time, it is a minor component (0.77% ± 0.61%) compared to the major brominated precursor tyrindoxyl sulphate (**2**). 6,6′-Dibromoindigo (**9**), better known as Tyrian purple, is the final dye pigment for many murex. The mass spectrum for this brominated dye pigment was consistent with a synthetic standard run by identical procedures and was identified at 13.9–14.1 min in LC-MS (Figure 4C) by triplet ion clusters at *m*/*z* 419, 421, and 423 (Figure 5D). Tyrian purple, which can be obtained by exposing tyriverdin (**8**) to sunlight, is well known to be insoluble in most solvents except in hot pyridine, DMF, or DMSO [7]. Several related but unidentified indoles were also detected in the chloroform extracts based on the UV and MS profiles (Supplementary Figure S2). These unknowns were minor constituents (Figure 4C) based on the relative percent composition of the extract (mean UB1 = 2.06% ± 0.65%, UB2 = 1.36% ± 0.54%, UB3 = 1.35% ± 0.70%).

It has been suggested that LC-MS may not be suitable for the identification of Tyrian purple pigments due to poor solubility in most solvents [38] and its long run time (usually 20 min to 2 h) [39]. This is an important consideration for archaeological research, but for modern applications the soluble precursors are more useful for the purpose of dyeing and for bioactive formulations.

### 2.2. Validation Studies

#### Precision, Linearity, Limit of Detection (LOD), and Limit of Quantification (LOQ)

The intra-day and inter-day relative standard deviations (RSD) were studied to evaluate the precision of the developed method using purified tyrindoxyl sulphate (**2**) as a standard for comparison. The RSD (*n* = 5) were 0.46% for the intra-day and 0.31% for the inter-day. According to the Food and Drug Administration, precision for all concentrations is acceptable if the % RSD falls within ±15% at each concentration level [40]. Therefore, the results demonstrate the precision of the method and so, its effectiveness for quantitative purposes.

Purified fractions of murexine (**1**), tyrindoxyl sulphate (**2**), and 6-bromoisatin (**6**) were used to generate standard curves (Supplementary Figure S3) that facilitated relative quantification (Table 2), using the developed method by adjusting the concentrations of the standards based on their purity (as explained in Section 3.4.2). The linearity of the analytical response within the concentration range is good for all three compounds, with correlation coefficients equal to 0.999, signifying linear relationships between the concentration of the standards and the corresponding peak area for absorbance at 210 nm (Supplementary Figure S3). The standard curves are reproducible and accurate for predicting the concentrations of the secondary metabolites in complex extract mixtures (Table 2). The standard curve for tyrindoxyl sulphate was also used to approximate the relative quantification of tyrindoxyl S-oxide sulphate (**3**), whereas 6-bromoisatin was used to estimate tyrindoleninone (**5**), tyriverdin (**8**), and 6,6′-dibromoindigo (**9**) after correcting the individual molecular weights of each compound.

The LODs are 0.029 mg/mL, 0.004 mg/mL, and 0.071 mg/mL for murexine, 6-bromoisatin, and tyrindoxyl sulphate, respectively. The LOQs are 0.087 mg/mL, 0.014 mg/mL, and 0.216 mg/mL for murexine, 6-bromoisatin, and tyrindoxyl sulphate, respectively. The LOQs for each of the reference standards are much higher than the respective LODs in all cases [41]. The LODs of the standards are higher than the reported LOD of tryptophan at 0.1 µg/mL [42]. This method could be applied to investigation of the biosynthetic pathway of the brominated indole precursors in Muricidae, to identify low levels of tryptophan and the biosynthetic intermediates that lead to tyrindoxyl sulphate. The brominated indoles are thought to be synthesised from indoles produced by symbiotic bacteria [43,44]. Bacteria are known to produce indoles from tryptophan through the action of a tryptophanase and/or other enzymes [45].

The calculated concentrations of murexine, tyrindoxyl sulphate, and 6-bromoisatin from the methanol, ethanol, and chloroform extracts are above their respective LODs, implying the reliability of the standard curves (Table 2). The concentrations of tyrindoleninone and tyriverdin were also above the LOD in methanol and chloroform extracts only and not for the ethanol (Table 2). Tyrindoxyl S-oxide sulphate is below the LOD for both methanol and ethanol extracts (Table 2). Finally, the concentration of 6,6′-dibromoindigo is above the LOD of 6-bromoisatin in chloroform (Table 2).

### 2.3. Yield of the Extracts and Quantification of the Main Precursors from Different Organic Solvents

The yields of the extracts of *D. orbita* samples, expressed as % recovery from the wet weight of the hypobranchial gland, were much higher in the alcoholic solvents, with mean % yield in a descending order for methanol > ethanol > chloroform (Table 2). Univariate analysis confirmed a strong effect of solvent on the yield (Pseudo F = 12.46; *p* = 0.0007). This implies that the total recovery of secondary metabolites is dependent upon the type of solvent used and this was largely driven by the recovery of the major polar components tyrindoxyl sulphate (logP − 0.35 [8]) and murexine (logP − 3.37 [8]), which were absent from chloroform extracts (Table 2). Tyrindoxyl S-oxide sulphate was also absent from the chloroform extract and, although it could be identified in the ethanol extract, it was beyond the LOD for accurate quantification (Table 2). Conversely, lipophilic 6,6′-dibromoindigo was only detected in the chloroform extracts (Table 2).

Table 2 shows the recovery of each analyte based on the standard curves for murexine, tyrindoxyl sulphate, and 6-bromoistain, as a proportion of the total weight of extract, as well as the initial weight of the wet tissue. One-factor permutational multivariate analysis (PERMANOVA) confirmed that there is a significant difference in the compound composition between the solvents (Pseudo F = 14.67, *p* = 0.003). Pair-wise tests revealed a significant difference in the composition of the alcoholic and chloroform extracts (methanol-chloroform *p* < 0.05 and ethanol-chloroform *p* < 0.05), but not between methanol and ethanol (*p* > 0.05). Canonical analysis on the principal coordinates (CAP) with trajectory overlay based on Spearman correlation (Figure 6) confirms that the polarity of the extraction solvents influences the composition of the extracts as expected based on the logP of the compounds (Table 2). The chloroform extracts separate along the *x* axis (CAP1, Figure 6), quite distinct from the alcohol extracts, due to the absence of hydrophilic ultimate precursors and more of the lipophilic compounds (logP > 1, Table 2, Figure 6), whereas the ethanol and methanol extracts separate along CAP2 (*y* axis, Figure 6) due to differences in the relative quantities of these precursors (Table 2). It is necessary to utilise prior knowledge of the chemical characteristics of the compounds, such as logP values [8,29], to maximise the extraction of the bioactive compound of interest using appropriate solvents.

### 2.4. Sample Stability Test

Tyrian purple precursors are known to easily degrade under certain circumstances. In order to check the stability of the extracts, stability testing of the extracts was performed by observing the changes in the relative peak areas of the dye precursor compounds as a percent of the total extract. For the first stability test, a multivariate PERMANOVA was used to compare extracts from freshly dissected snails (S1) and snails that were frozen for 1 month prior to extraction (S2). Results showed that the composition of the extracts from the fresh and frozen snails was not statistically different (*p* > 0.05), indicating that short-term freezing did not alter the relative abundance of the secondary metabolites in the snails as long as they are properly stored, such as keeping the samples in a −20 °C or −80 °C freezer with very minimal light and oxygen exposure (Figure 1B).

**Figure 7:**
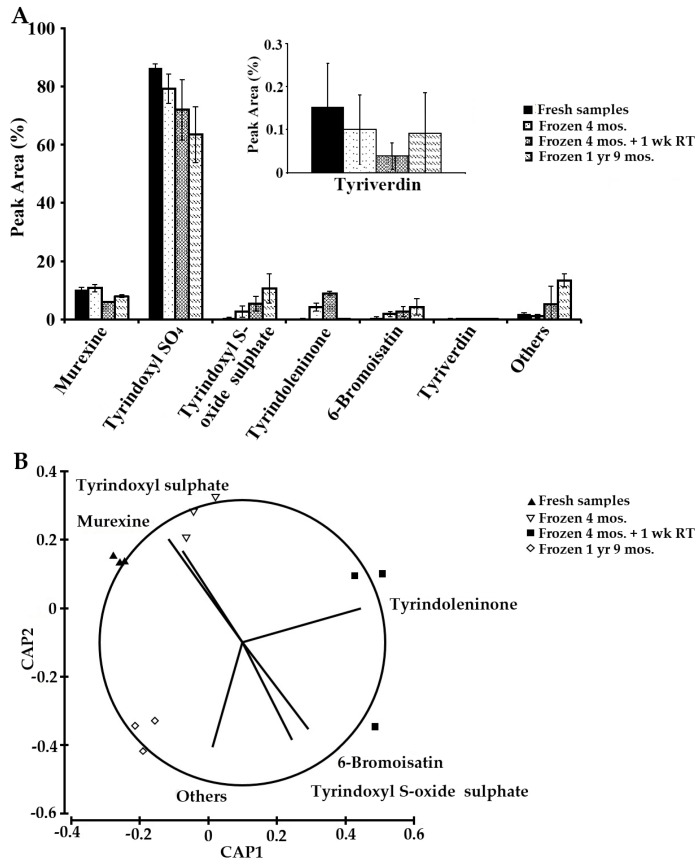
Stability of the bioactive compounds from ethanol extracts of the hypobranchial gland of *Dicathais orbita* (*n* = 3). (**A**) Histogram showing the relative (%) integrated peak area from HPLC absorbance values at 210 nm for the bioactive compounds of *D. orbita* analysed at different times. Inset graph shows magnification of the changes in tyriverdin; (**B**) Canonical analysis on the principal coordinates with vector overlay based on Spearman correlation > 0.6.

The subsequent stability tests, however, gave a different result. Fresh extracts (S1) were compared to extracts after four months of storage in the freezer after sealing in N_2_ gas (S3 = no light or air) and then the same samples were left at room temperature in typical laboratory conditions for light and air exposure for another week (S4). The same extracts were stored again in the freezer and rerun after one year and nine months of cold storage (in the dark) (S5). Multivariate PERMANOVA analysis revealed that the composition of the ethanol and chloroform extracts was significantly affected by long-term cold storage (*p* < 0.05). In the ethanol extracts, the oxidative products 6-bromoisatin (**6**) and tyrindoxyl S-oxide sulphate (**3**) increased with time (Figure 7A). Tyrindoxyl S-oxide sulphate showed a significant increase in terms of peak area after months of cold storage (*p* < 0.05). Conversely, the tyrindoxyl sulphate (**2**) was found to decrease after months of storage and exposure to light (Figure 7A). 6-Bromoisatin is a by-product of the decomposition of tyriverdin (**8**), which may explain the increase in the amount of 6-bromoisatin coinciding with the decrease of tyriverdin after four months storage and one week at room temperature (Figure 7A). As can be seen in the CAP plot (Figure 7B), all samples with the same period of storage are clustered together with S1 and S3 characterised by the ultimate precursors murexine and tyrindoxyl sulphate, while S4 is characterised by higher oxidation products 6-bromoisatin and tyrindoxyl S-oxide sulphate, as could be expected from exposure to air and light on a standard laboratory bench. S5 extracts, after long-term storage, have more of the other unaccounted compounds, indicating a clear progression in the chemical degradation of the compounds.

**Figure 8:**
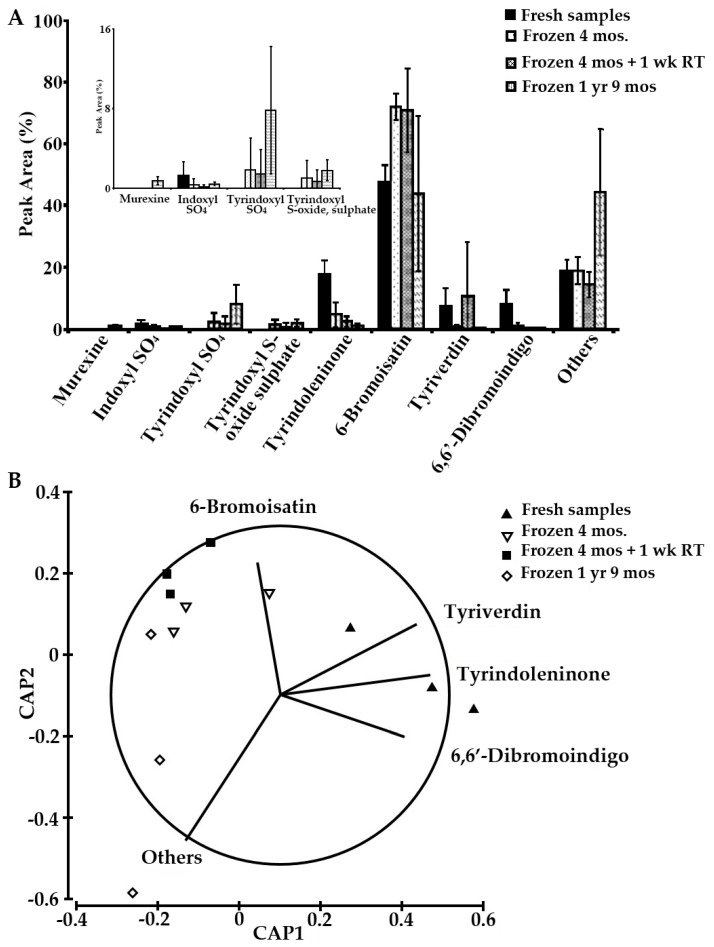
Stability of the bioactive compounds from chloroform extracts of the hypobranchial gland of *Dicathais orbita* (*n* = 3). (**A**) Histogram showing the relative (%) integrated peak areas from the HPLC absorbance at 210 nm of the bioactive compounds of *D. orbita* analysed at different times. Inset graph shows magnification of the changes in minor compounds; (**B**) Canonical analysis on the principal coordinates with vector overlay based on Spearman correlation > 0.6.

The chloroform extracts showed higher variability in the secondary metabolite composition and have more degradation products (Figure 8). Murexine (**1**), tyrindoxyl sulphate (**2**), and tyrindoxyl S-oxide sulphate (**3**), which were initially not detected in the fresh extracts, were detected in very small amounts as the extracts were stored for longer (Figure 8A). Samples stored for a much longer term (S5) are characterised by more of the degradation products, which seem to double as a relative percent of the extract (Figure 8A) in comparison to ethanol extracts (Figure 7A). Separation along the *x* axis in the CAP plot distinguishes the fresh samples, which have more tyrindoleninone (**5**), tyriverdin (**8**), and 6,6′-dibromoindigo (**9**); while, after freezing, there was more of the 6-bromoisatin (Figure 8B). As 6,6′-dibromoindigo is a highly stable compound, the drop after freezing and storage is likely due to oxidation of precursors prior to analysis, as well as the high insolubility of the compound due to intramolecular bonding after it forms. Thus 6,6′-dibromoindigo is likely to be under-represented in our analyses. Nevertheless, the results suggest the potential for oxidative degradation of muricid extracts and imply that they should preferably be analysed fresh after extraction or stored for the minimal amount of time in a freezer for comparative analysis and use as nutraceuticals.

Overall, the results of this study show that ethanol is a preferentially effective solvent for the extraction of the ultimate precursors of Tyrian purple found in *D. orbita*, with high reproducibility and fewer artefacts from post-extraction degradative reactions than found in chloroform and methanol. This reproducibility is also evident in the different dilutions made for the ethanol extracts (Supplementary Table S1). The efficiency of the extraction method is also an important point to consider as macerating the hypobranchial gland prior to the addition of the solvent may introduce more degradation and potential artefacts to the samples compared to macerating the gland concurrently with the solvent (Supplementary Figure S4). Once extracted, the ultimate precursor tyrindoxyl sulphate can be converted into any of the intermediates or final dye pigments by controlling the oxygen and light conditions after hydrolysis using an aryl sulphatase enzyme. If one needs to recover the intermediate precursors of the dye, then chloroform may be a better choice of solvent. However, if a more realistic picture of what molluscs produce in their tissues is needed, alcohol is preferred. The choice of extraction solvent, therefore, plays a key role in the experimental design and has to be chosen carefully depending on the aim of the extraction and the target compounds. To meet the requirements of GMP for safe routine handling and for potential therapeutic applications, ethanol is recommended over other extraction solvents as it is less toxic, yet an effective extraction medium.

## 3. Materials and Methods

### 3.1. Collection of Samples

A total of 50 *D. orbita* individuals were collected by hand from the intertidal reefs of Shelly Beach, Ballina (28°51′56.6″ S 153°35′39.1″ E) and Evans Head (29°08′06.2″ S, 153°27′00.0″ E), New South Wales, Australia between March to December 2014 and August 2016 under NSW Fisheries permit numbers F89/1171-6.0 and P10/0069-1.0. The snails were transported to Southern Cross University, Lismore, NSW, Australia, in fresh seawater obtained from the sampling site. The collected snails were used for the comparison of different extraction solvents, as well as ESI and CI methods in mass spectrometry and for validation studies.

### 3.2. Dissection and Extraction

The dissection of the hypobranchial gland of *D. orbita* followed the methods of Westley and Benkendorff [21]. Briefly, the shell of the snail was removed by rupturing it cautiously with a bench-top vice. The hypobranchial gland was removed by excision along the junction between the gland and the gill tissue with surgical scissors, and separation from the shell margin and surrounding tissue. Glands were weighed on an analytical balance (Mettler Toledo ML 204 Greifensee, Switzerland, with 0.001 g precision) to obtain the extract weight per wet weight of tissue.

Sample preparation is the most important step in the development of methods for the standardised analysis of botanicals and herbal preparations [46]. In this study, two extraction methods were employed. One was the homogenisation of the gland together with the solvent and the other procedure involved macerating the gland before the addition of the solvent (denoted hereinafter as E1 and E2, respectively, Supplementary Figure S4).

#### Extraction Method

After dissection, each gland was homogenised using a mortar and pestle with at least 10 mL of solvent. Extractions were performed using either analytical grade methanol, ethanol, or chloroform (Sigma-Aldrich, Castle Hill, Australia). The homogenised gland was transferred to a glass vial. The mortar and pestle were rinsed with additional solvent and combined with the gland mixture to give 15 mL solvent in total. The vial containing the homogenised mixture was covered with aluminium foil to prevent photolytic degradation of the metabolites and set aside overnight in a cold room at −4 °C. The mixture was then filtered using Sigma-Aldrich Whatman filter paper grade 1. Resulting extracts were dried under vacuum (Vacuum controlled, V-800 and V-500, rotary evaporator, rotavapor R-114; Buchi Labortechnik AG, Flawil, Switzerland) at 40 °C, giving an oily reddish brown residue. Dried extracts were redissolved in minimal solvent for transfer into pre-weighed vials, further dried under nitrogen gas (N_2_), and weighed for extraction efficiency. Samples from each selected solvent were protected from light and stored at −20 °C prior to analysis. The vacuum pressure during rotary evaporation for each solvent was maintained as follows: methanol, 337 mbar Hg; ethanol, 175 mbar Hg; and chloroform, 474 mbar Hg. Ethanol was selected to further investigate the stability of the bioactive compounds as described in Section 2.4 and for the comparison of extracts using LC-MS-ESI and CI methods in positive and negative modes.

### 3.3. LC-MS Analysis

The dried extracts were analysed by liquid chromatography-mass spectrometry (LC-MS) using an Agilent (Santa Clara, CA, USA) 1260 infinity high-performance liquid chromatography (HPLC) system coupled with a 6120 Quad mass spectrometer. The HPLC system consisted of a diode array detector (G4212B), binary pump (G4220A), an autosampler (G4226A), a vacuum degasser, and a column oven. Each extract was run using the mass spectrometer interfaced with electrospray ionisation (ESI) and chemical ionisation (CI) methods operated in both positive and negative ion modes. The chromatography was performed on a Phenomenex (Lane Cove, Australia) luna C18 (2) HPLC column (100 mm × 4.6 mm) using Milli-Q water (Merck Millipore, Bayswater, Vic, Australia) and acetonitrile (ACN, Scharlau supragradient HPLC grade) with 0.005% trifluoroacetic acid (TFA, Sigma-Aldrich, Castle Hill, Australia). The optimal solvent gradient for separation was 20% acetonitrile (ACN) in water and was increased to 95% ACN over 18 min, at a flow rate of 0.5 mL/min and an injection volume of 5 µL. The extracts were reconstituted with 2 mL methanol before injecting in the machine. The ethanol extracts were further diluted with 5 and 10 mL methanol to determine which will give the most optimal concentration of the precursors.

Using the Agilent ChemStation software (2000-2-11, Agilent Technologies, Mulgrave, Vic, Australia)), principal compounds within the extracts were identified using ESI with supplementary analysis using CI and identified by their characteristic major doublet (singly brominated) or triplet (doubly brominated) mass ion clusters for the brominated indoles [21]. Specific screening for 224/226 ions was made as these are the major fragment ions for the bromoindoles. Parallel UV/Vis diode-array detection (DAD) was used at 200–600 nm. For routine comparisons and quantification of murexine, 6-bromoisatin, and tyrindoxyl sulphate, compounds were monitored at 210 nm.

### 3.4. Identification of the Secondary Metabolites

The identification of secondary metabolites was carried out by comparing their retention times and mass-to-charge ratio to previous literature. Reference standards of pure synthetic 6-bromoisatin (TCI AMERICA, Portland, OR, USA) and the purified murexine and tyrindoxyl sulphate were also run under the same LC-MS conditions for comparison. The latter two standards were isolated on preparative HPLC system and identity confirmed by ^1^H-nuclear magnetic resonance (^1^H-NMR) and high-resolution mass spectrometry (HR-MS) as described below.

#### 3.4.1. Purification of Reference Standards

As only 6-bromoisatin was available for use as a synthetic standard, preparative HPLC was employed to separate murexine and tyrindoxyl sulphate from the pooled ethanol extracts of the hypobranchial glands (*n* = 15) from *D. orbita*. An Agilent (Santa Clara, CA, USA) 1260 Infinity-HPLC preparative system equipped with a C-18 Phenomenex preparative column (150 mm × 21.20 mm × 5 µm packing; 100 Å) was used with a solvent system comprising of ACN and Milli-Q water with 0.05% TFA added for each solvent. The gradient program started with 18% ACN and then was increased linearly up to 95% in 13 min and then remained constant for 5 min, before returning to 18%, until a total run-time of 20 min was reached. The column flow was 15 mL/min and the injection volume was 250 µL. Murexine was collected by an Agilent Tech 1260 Infinity fraction collector between 2 and 3.2 min using consecutive time slices every 0.2 min, whereas tyrindoxyl sulphate was collected between 7 and 8.5 min using time slices every 0.3 min.

The isolated fractions were dried using a rotary evaporator and further concentrated under a stream of 100% nitrogen gas and then finally freeze-dried (Alpha 2–4; Christ, Osterode am Harz, Germany). To neutralise TFA, a small amount of ammonia (Univar, Ajax Finechem, Sydney, Australia) was added prior to drying the tyrindoxyl sulphate fraction. Unlike murexine, which is resistant to acid hydrolysis [18], tyrindoxyl sulphate can undergo hydrolysis in the presence of an acid to form the dye end products [7]. To demonstrate this problem, chromatograms showing the purified tyrindoxyl sulphate fraction dried in a rotary evaporator with and without ammonia are presented in Supplementary Figure S5.

#### 3.4.2. Confirmation of Reference Compound Identification

To further confirm the identification of the purified murexine and tyrindoxyl sulphate, samples were analysed by ^1^H-NMR and HR-MS. ^1^H-NMR spectra were recorded on a Bruker Avance III HD 500 MHz spectrometer (Bruker Biospin, Alexandria, Australia) in CD_3_CN and D_2_O (Novachem, Cambridge Isotopes Laboratories, Tewksbury, MA, USA). ^1^H chemical shifts were referenced to either ACN-δ6 (1.96 ppm) or D_2_O (4.80 ppm). The ^1^H-NMR assignments for murexine and tyrindoxyl sulphate are presented in Table 1. These are consistent with previously published ^1^H-NMR reports for these compounds [47].

HR-MS was obtained on a Bruker 12 T SolariX XR FT-ICR mass spectrometer (Bruker Daltonics, Preston, Vic, Australia). This confirmed a peak with *m*/*z* at 224.139686 with 100% match to the molecular formula C_11_H_18_N_3_O_2_ consistent with murexine. The tyrindoxyl sulphate fraction contained peaks matching the [M + H, Br^79^]^+^ at 337.914597 100% score for C_9_H_9_BrNO_4_S_2_ and [M+ Na, Br^79^]^+^ at 359.896482 C_9_H_8_BrNNaO_4_S_2_. Detection of the [M + H] ion is consistent with previous MS imaging studies on *D. orbita* hypobranchial gland tissue using desorption ionisation on porous silicon (DIOS) [47] and nano-assisted laser desorption/ionisation-MS (NALDI-MS) [29] to detect ions in the low molecular mass range. The tyrindoxyl sulphate fraction also contained some murexine, choline and brominated degradation products, but was nevertheless the dominant compound in this fraction.

Murexine was further confirmed by thin layer chromatography (TLC), where a pink-coloured pigment was formed after spraying with Dragendorff reagent according to the methods of Roseghini et al. [18]. Dragendorff reagent is used to visualise alkaloids and quaternary ammonium ions such as choline and its esters that have been separated using TLC [18]. TLC was run on an aluminium-backed silica gel plate (TLC Silica Gel 60 F254, Merck Millipore, Bayswater, Vic, Australia) with *n-*butanol–ethanol–acetic acid–water (8:2:1:3) (Sigma-Aldrich, Milli-Q) solvent system.

The purity of compounds was calculated as the % of all peaks in the HPLC at 210 nm from the highest concentrations used in the standard curves. Murexine easily breaks down to form choline after purification and the estimated purity for murexine was 93.20%. The purity of the tyrindoxyl sulphate obtained was 96.30%, and the synthetic 6-bromoisatin standard was 90.02%. The concentrations were adjusted accordingly for the respective calibration curve of the standards.

### 3.5. Method Validation: Precision, Linearity Curve, Limit of Detection (LOD), and Limit of Quantitation (LOQ)

The intra-day precision was evaluated by analysing the peak under the curve of tyrindoxyl sulphate in triplicate on a single day, while the inter-day precision was carried out over a period of three days.

The desired concentration range for each compound was obtained by serial dilution for standard curve preparation. The samples were analysed by LC-MS and the area under the peak (monitored at 210 nm) versus the concentrations of the standard was tested by linear least square regression analysis. Each standard calibration curve was generated using ten data points, covering the concentration ranges 0.005–10.014 mg/mL, 0.004–1.886 mg/mL, and 0.002–20.897 mg/mL for murexine, 6-bromoisatin, and tyrindoxyl sulphate, respectively. Injections were performed in triplicate for each concentration.

The LOD for tyindoxyl sulphate was calculated to be 3 times the standard deviation of the intercept and the slope of the calibration curve, where the slope was estimated from the calibration of the analyte. The LOQ was calculated from 10 times the standard deviation value.

### 3.6. Stability Testing

The stability of the samples was ascertained by comparing the changes in the composition and quantity of the bioactive compounds from the fresh and frozen snails. The first stability test was the comparison between the fresh and frozen snails. Fresh samples (S1) used live snails extracted immediately after collection from the field with ethanol (*n* = 3) and chloroform (*n* = 3). The frozen samples (S2) utilised snails (*n* = 3) that were stored in a −20 °C freezer for a month prior to extraction. The second stability test was to compare the composition of fresh and frozen extracts after longer term storage. After injection in the LC-MS, the same S1 samples (*n* = 3) above were stored in −80 °C freezer for four months (referred to as S3) and then re-analysed by LC-MS. Then these samples (*n* = 3) were left at room temperature in the laboratory and exposed to standard conditions of air and light for another week and re-analysed by LC-MS; these extracts are referred to as S4. After one year and nine months of cold storage (S5), the same samples were injected again in LCMS.

### 3.7. Statistical Analysis

Statistical analyses were conducted in PRIMER v6 + PERMANOVA add-on (Plymouth, UK). Univariate PERMANOVA analyses were used to assess the effects of solvent on the yield of the extracts using three different organic solvents, whereas multivariate analyses were used to compare the compound composition using different solvents, extraction methods, and storage treatments. Canonical analysis on the principal coordinates (CAP) with vector overlay based on Spearman correlation >0.6 was used to graphically represent differences of the secondary metabolite composition according to solvent extracts, as well as to illustrate the significant changes due to storage of the extracts. Post hoc pair-wise tests were run to further explore which treatment groups were significantly different. *p* values less than 0.05 were reported as statistically significant in all analyses. Data are expressed as means ± standard deviation.

## 4. Conclusions

In summary, ethanol was identified as a suitable solvent for extracting the ultimate precursors of Tyrian purple by homogenising Muricidae hypobranchial gland tissue in the solvent. LC-ESI-MS in both positive and negative detection modes with complementary UV-DAD spectra provides more information useful for the qualitative and quantitative assessment of the bioactive choline esters and brominated indole derivatives from *D. orbita*. It is likewise important to note that extraction solvents, storage methods, ionisation methods, and ion detection modes in the mass spectrum all influence the extract composition and ability to detect bioactive compounds from Muricidae molluscs, and thus a standardised method is required. Despite the wide structural diversity and polarity of the compounds analysed, only a simple extraction protocol in ethanol was required. The LC-MS method proved to be efficient for the quantification of both classes of bioactive secondary metabolites investigated, as demonstrated by the precision, linearity, LOD, and LOQ. The ability to switch between ESI and CI as well as positive and negative ion modes is also a significant advantage. For promising nutraceuticals, a well-validated method will help establish the quality of extracts and facilitate future approval as a therapeutic agent for human use.

## Figures and Tables

**Table 1:** ^1^H-NMR assignment for murexine (CD_3_CN)) and tyrindoxyl sulphate (D_2_O) isolated from the hypobranchial gland extract of *Dicathais orbita* by HPLC preparative system.

Compound	δ (ppm)	Multiplicity	Relative Area	Assignment
1. Murexine	3.18	s	9	1
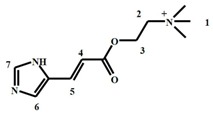	3.62–3.68	m	2	2
	4.53–4.68	m	2	3
	6.62	d	1	4
	7.68	d	1	5
	7.50	s	1	6
	7.92	s	1	7
2. Tyrindoxyl sulphate	2.50	s	3	1
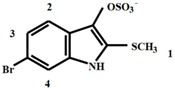	7.20	dd	1	2
	7.55	d	1	3
	7.65	d	1	4

**Table 2:** Yield of solvent extracts (% *w*/*w*) from the hypobranchial glands *D. orbita* and the mean (±SD) weight of precursor compound, as a proportion of the total extract weight (mg/mg) and weight of wet hypobranchial tissue prior to extraction (alcoholic extracts: *n* = 4; chloroform extracts: *n* = 7).

% Yield	logP ^1^	Methanol		Ethanol		Chloroform	
		17.53 ± 3.14 %g/g		12.20 ± 3.17 %g/g		7.00 ± 3.52 %g/g	
		Dry Extract (mg/mg)	Wet Tissue (mg/mg)	Dry Extract (mg/mg)	Wet Tissue (mg/mg)	Dry Extract (mg/mg)	Wet Tissue (mg/mg)
(**1**) Murexine	−3.37	0.431 ± 0.175	0.087 ± 0.023	0.468 ± 0.275	0.071 ± 0.024	n.d.	n.d.
(**2**) Tyrindoxyl sulphate	−0.35	0.309 ± 0.124	0.062 ± 0.016	0.293 ± 0.167	0.044 ± 0.014	n.d.	n.d.
(**3**) Tyrindoxyl S-oxide sulphate ^2^	-	0.005 ± 0.002	0.001 ± 0.001	<LOD	<LOD	n.d.	n.d.
(**5**) Tyrindoleninone ^3^	2.89	0.004 ± 0.002	0.001 ± 0.001	0.003 ± 0.002	0.001 ± 0.000	0.018 ± 0.004	0.001 ± 0.001
(**6**) 6-Bromoisatin	1.62	0.006 ± 0.002	0.001 ± 0.000	0.005 ± 0.003	0.001 ± 0.000	0.044 ± 0.015	0.003 ± 0.001
(**8**) Tyriverdin ^3^	4.67	0.004 ± 0.002	0.001 ± 0.000	0.003 ± 0.001	>0.001 ± 0.000	0.035 ± 0.014	0.002 ± 0.000
(**9**) 6,6′-Dibromoindigo ^3^	4.47	n.d.	n.d.	n.d.	n.d.	0.031 ± 0.017	0.002 ± 0.001

^1^ logP is a solubility indicator based on octanol-water partition coefficient calculated using the chemoinformatics software Molinspiration; ^2^ Estimated from tyrindoxyl sulphate standard curve; ^3^ Estimated from 6-bromoisatin standard curve.

## Supplementary Materials

Supplementary materials can be accessed at: http://www.mdpi.com/1420-3049/21/12/1672/s1.
