# Supplementary material for: Extraction and Quantification of Bioactive Tyrian Purple Precursors: A Comparative and Validation Study from the Hypobranchial Gland of a Muricid Dicathais orbita
Source: Molecules. 2016 Dec 5;21(12):1672. doi: 10.3390/molecules21121672 (PMC6273837; doi:10.3390/molecules21121672)
Supplement: Supplementary file 1 [file molecules-21-01672-s001.pdf]

Supplementary Materials: Extraction and Quantification of Bioactive Tyrian Purple Precursors: A Comparative and Validation Study from the Hypobranchial Gland of a Muricid *Dicathais orbita*

Roselyn Valles-Regino, Peter Mouatt, David Rudd, Lachlan H. Yee, and Kirsten Benkendorff

Table S1. Mean (±SD) weight of precursor compound as a proportion of the total extract weight (mg/mg) and weight of wet hypobranchial tissue prior to extraction ($n = 4$).

Compound	Dry Extract (mg/mg)		Wet Tissue (mg/mg)	
	**5 mL**	**10 mL**	**5 mL**	**10 mL**
(**1**) Murexine	0.317 ± 0.197	0.213 ± 0.148	0.047 ± 0.018	0.031 ± 0.014
(**2**) Tyrindoxyl sulphate	0.179 ± 0.113	0.126 ± 0.089	0.026 ± 0.010	0.018 ± 0.009
(**3**) Tyrindoxyl *O*-sulphate	<LOD	<LOD	<LOD	<LOD
(**5**) Tyrindoleninone	0.006 ± 0.002	0.007 ± 0.000	0.001 ± 0.000	0.001 ± 0.000
(**6**) 6-Bromoisatin	0.008 ± 0.005	0.008 ± 0.004	0.001 ± 0.000	0.001 ± 0.000
(**8**) Tyriverdin	0.001 ± 0.000	<LOD	<0.001 ± 0.000	<LOD

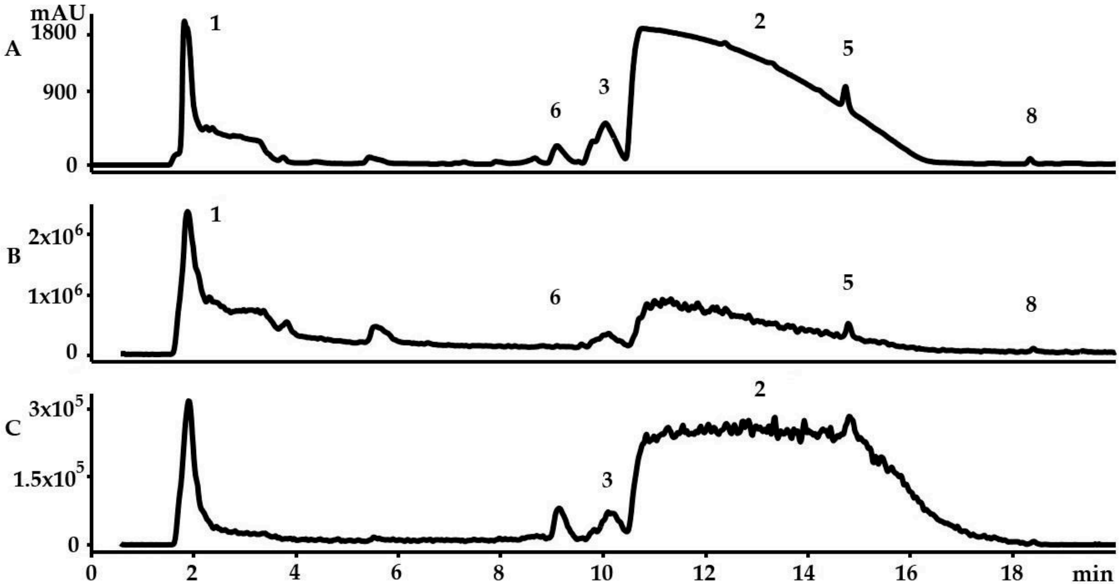

Figure S1. Representative chromatograms of the ethanol extract from the hypobranchial gland of *Dicathais orbita*: (**A**) UV-Vis spectra; (**B**) positive and; (**C**) negative chemical ionisation (CI) mass spectra (TIC) showing the bioactive compounds of interest. Peak assignments based on Figure 1 are as follows: (**1**) murexine, (**2**) tyrindoxyl sulphate, (**3**) tyrindoxyl S-oxide sulphate, (**5**) tyrindoleninone, (**6**) 6-bromoisatin, and (**8**) tyriverdin.

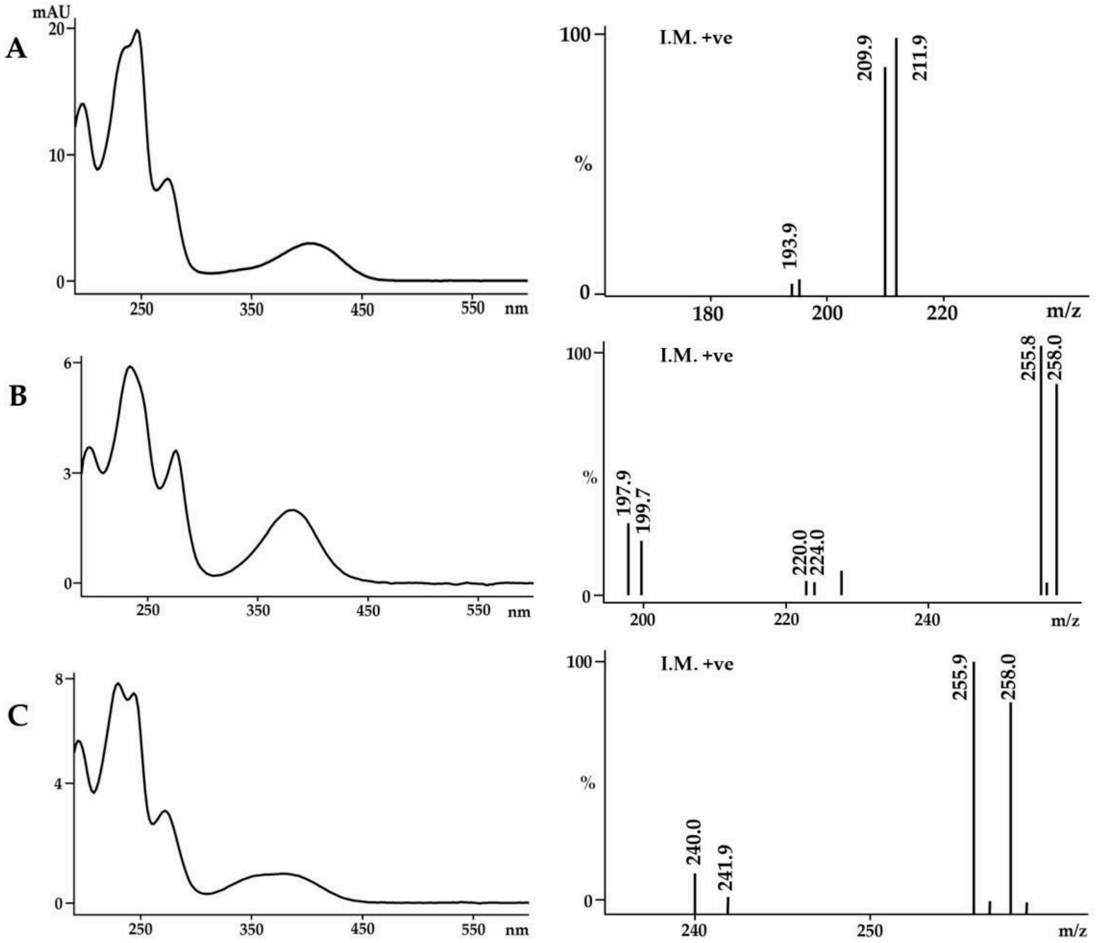

Figure S2. UV spectra (**left**) with mass spectra (**right**) of unidentified brominated compounds (**A**) UB1; (**B**) UB2; and (**C**) UB3 obtained from bioactive compounds found in the hypobranchial gland chloroform extract of *Dicathais orbita* from high-performance liquid chromatography–mass spectrometry with electrospray ionisation. I.M. ionisation mode: I.M. +ve = positive ions.

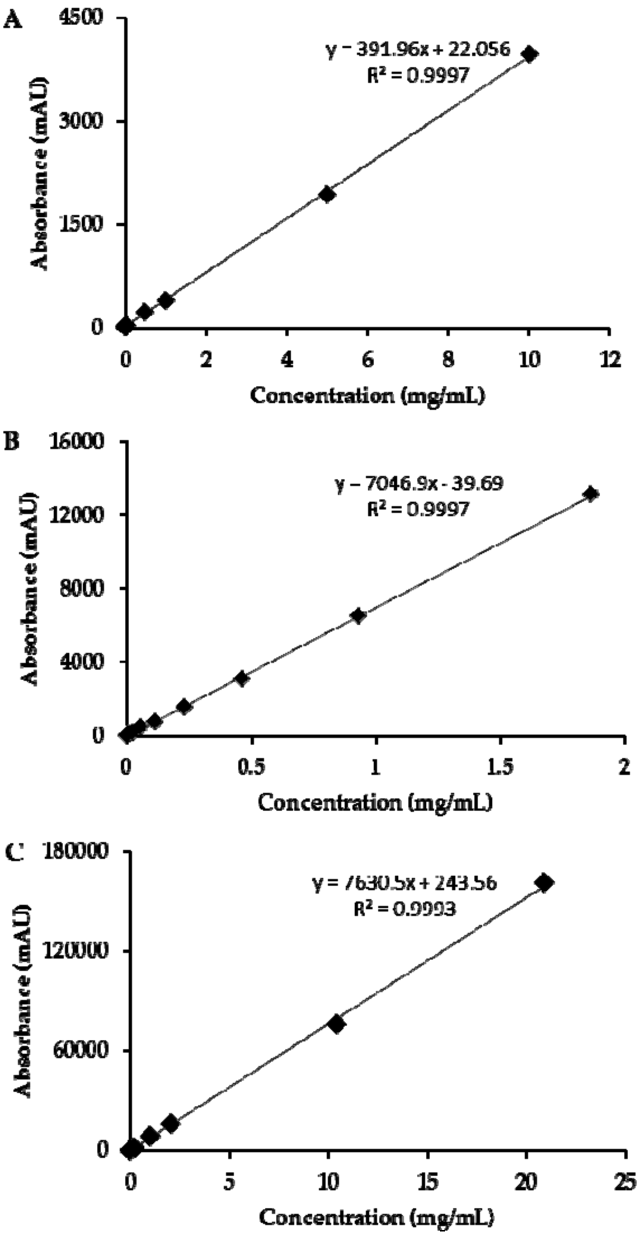

Figure S3. Calibration curves of (**A**) murexine; (**B**) 6-bromoisatin; and (**C**) murexine monitored at 210 nm.

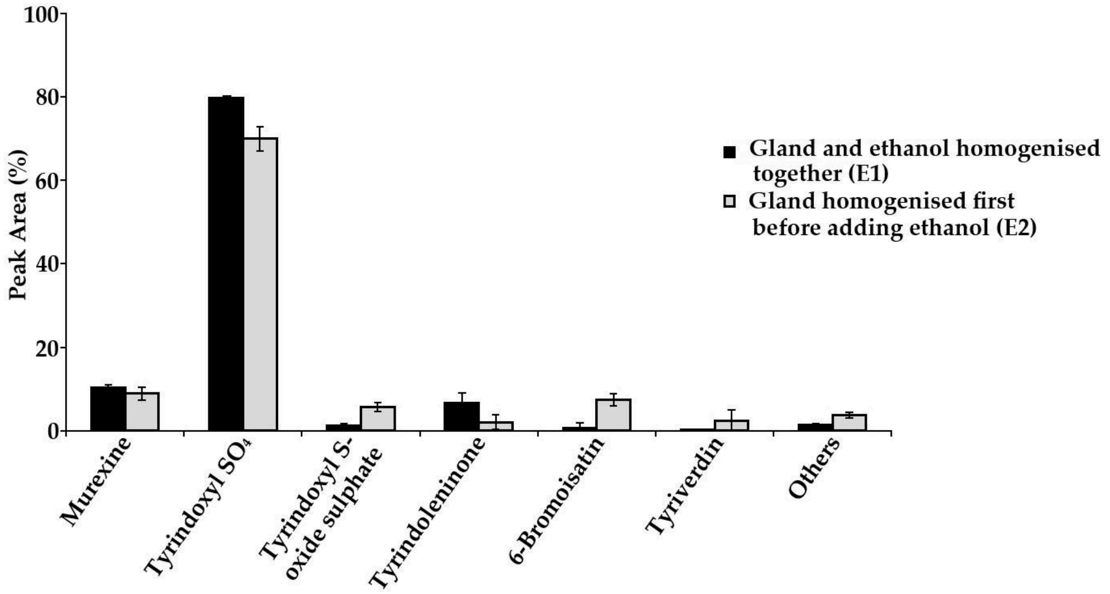

Figure S4. Comparison of the composition of the secondary metabolites in *Dicathais orbita* extracts prepared using two different extraction methods. Compounds were identified by high-performance liquid chromatography–mass spectrometry with electrospray ionisation and quantified by relative absorbance at 210 nm.

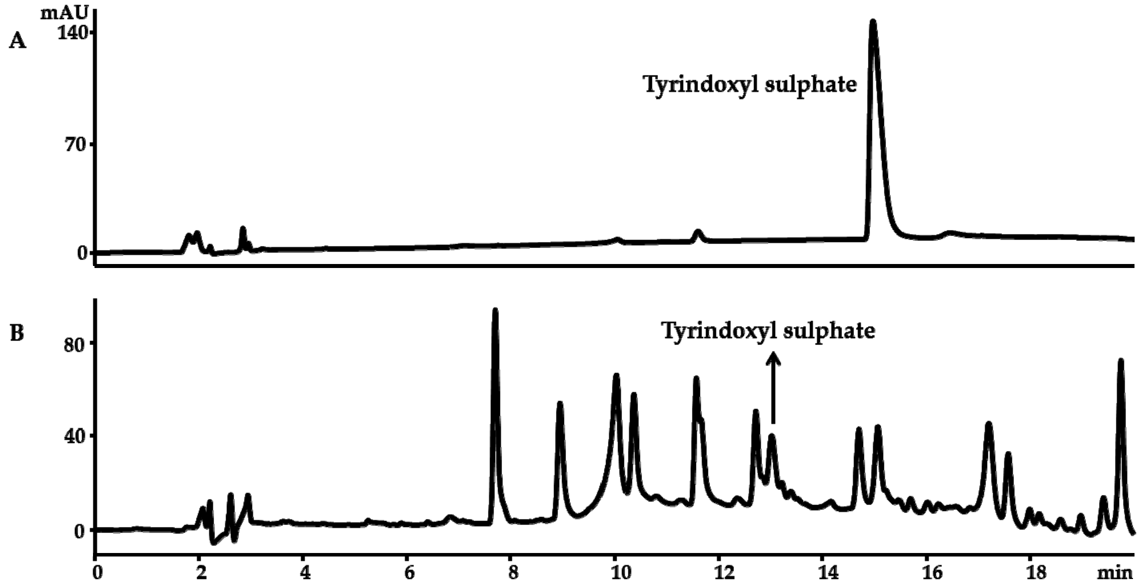

Figure S5. Chromatograms of the isolated tyrindoxyl sulphate from the hypobranchial gland of *Dicathais orbita* based on retention time using the high-performance liquid chromatography preparative system: (**A**) Tyrindoxyl sulphate fraction that was dried in rotary evaporator with the addition of ammonia; (**B**) tyrindoxyl sulphate fraction dried in rotary evaporator without ammonia.
